# Designing stepping-stones landscapes: a 2D perspective does not lead to more standardization than an in-situ perspective

**DOI:** 10.3389/fpsyg.2024.1360198

**Published:** 2024-02-26

**Authors:** Amy M. Jeschke, Simone R. Caljouw, Frank T. J. M. Zaal, Rob Withagen

**Affiliations:** Department of Human Movement Sciences, University Medical Center Groningen, University of Groningen, Groningen, Netherlands

**Keywords:** 2D map-based design, in-situ design, nonstandardization, playgrounds, stepping-stones landscapes

## Abstract

Previous research found that when participants across the lifespan could be the architect of their own stepping-stones landscapes, they create nonstandardized configurations with gap-width variation. Yet, architects often use standardized dimensions in their designs for playgrounds and outdoor fitness areas. To scrutinize why architects tend to seek for more standardized designs than the examined target users, we tested the hypothesis that the difference is caused by a different perspective during the making process. After all, landscape architects generally design on 2D maps, while the participants designed in situ. We asked 67 participants to design a stepping-stones landscape on a 2D map and 67 other participants to create the landscape in situ. Contrary to our expectations, we found no indications that designing on a 2D map leads to more standardized configurations. We end with discussing other characteristics of the design processes that could potentially explain the omnipresent standardization in design.

## 1 Introduction

Although playgrounds and outdoor fitness areas have the potential to counteract physical inactivity (e.g., Kahn et al., 2002; Colabianchi et al., 2009; Talarowski et al., 2019; Cohen et al., 2020; Zhai et al., 2020), research on stepping-stones landscapes suggests that the predominant standardized dimensions are not in line with the target users’ preferences (Jongeneel et al., 2015; Sporrel et al., 2017; Jeschke et al., 2020, 2022). On the one hand, architects often create standardized playgrounds and outdoor fitness areas, where components (e.g., bars, steps, rings etc.) are equally sized and the distances between them equally spaced. For example, the influential architect Aldo van Eyck generally organized his famous stepping-stones landscapes in a standardized, symmetrical figure-eight pattern, creating only two different gap widths to cross (Withagen and Caljouw, 2017). However, on the other hand, a series of experiments revealed that children, young and older adults create stepping-stones landscapes with variation in gap widths (Sporrel et al., 2017; Jeschke et al., 2020) and judge landscapes with nonstandardized dimensions as more fun and even as more esthetically appealing than the standardized ones (Sporrel et al., 2017; Jeschke et al., 2022, 2023).^1^ Hence, to help architects to arrive at designs that meet the desires of the target users, it is important to scrutinize why participants did not seek for standardization, while architects generally do.

One hypothesis is that the different pull toward standardization could be explained by characteristics of the design processes. That is, while the participants in the reported studies designed the landscapes in situ (Jongeneel et al., 2015; Jeschke et al., 2020), landscape architects traditionally design on a map. Already in 1990, Olwig suggested that this might make a difference.

The transferral of environmental information to a plane surface itself represents a form of abstraction that is biased against the environmental experience and preferences of children. To begin with, the plan, map, or blueprint gives priority to visual information and can be expected to foster a professional discourse that gives primacy to space and to visual forms and appearances. Geographers are thus often prone to define their field as “a spatial science,” whereas the reading of most any architectural journal will reveal a bias to the language of visual form (Olwig, 1990, p. 48).

And although nowadays 3D software is widely available, Li et al. (2014) found that most members of the American Society of Landscape Architects still design on 2D maps—only 30% of the 427 responders often used 3D software during the design process. Thus, it could be that architects tend to seek for more standardized designs than the individuals who participated in earlier research (Jongeneel et al., 2015; Sporrel et al., 2017; Jeschke et al., 2020, 2022), because architects often design on a plane surface, while these participants designed the configurations In Situ.

We tested this hypothesis in the present study. To that end, we analyzed stepping-stones landscapes created by participants in two different conditions. One group of participants was to create the configuration on a grass plot (in-situ group) whereas another group was to create the landscape on a computer that displayed a 2D map of the field (map group). We compared the number of different gap widths created from either perspective, to determine if the configurations created by the map group were indeed more standardized.

## 2 Method

### 2.1 Participants

A priori power analysis using G*Power version 3.1 (Faul et al., 2007) indicated that a minimum sample size of 64 persons per group was needed to detect a significant medium effect (*d* = 0.50; see Cohen, 1988) with α = .05 and a power of 80% for an independent samples *t*-test. Participants were eligible for inclusion if they did not have any physical trauma that could influence their jumping capabilities nor heard of the stepping-stones research results before. Participants were students and members of the public with no background in architecture, recruited via advertisements at the University of Groningen and several public places (e.g., supermarkets, student associations, and sport clubs). Participants were randomly allocated to either the map or in-situ group. Two participants were excluded from the data analysis because they did not fully comply with the given instructions. The remaining 134 participants (18–54 years old) were included in the data analysis. The anthropometrics of both groups are presented in Table 1. Independent samples *t-*test showed no significant difference between groups regarding their age (*t*(132) = −0.637, *p* = .525, *d* = 0.110) or leg length (*t*(132) = −0.437, *p* = .663, *d* = 0.075). The study was approved by the local ethics committee and all participants gave informed consent.

**Table 1:** The anthropometrics (means and standard deviations) of the participants in both groups.

Group	*N*	Gender	Age (years)	Leg length (cm)
Map	67	48 F/19 M	23.5 ± 5.1	86.7 ± 6.2
In Situ	67	46 F/21 M	24.0 ± 5.4	87.1 ± 5.7

### 2.2 Procedure

**Figure 1:**
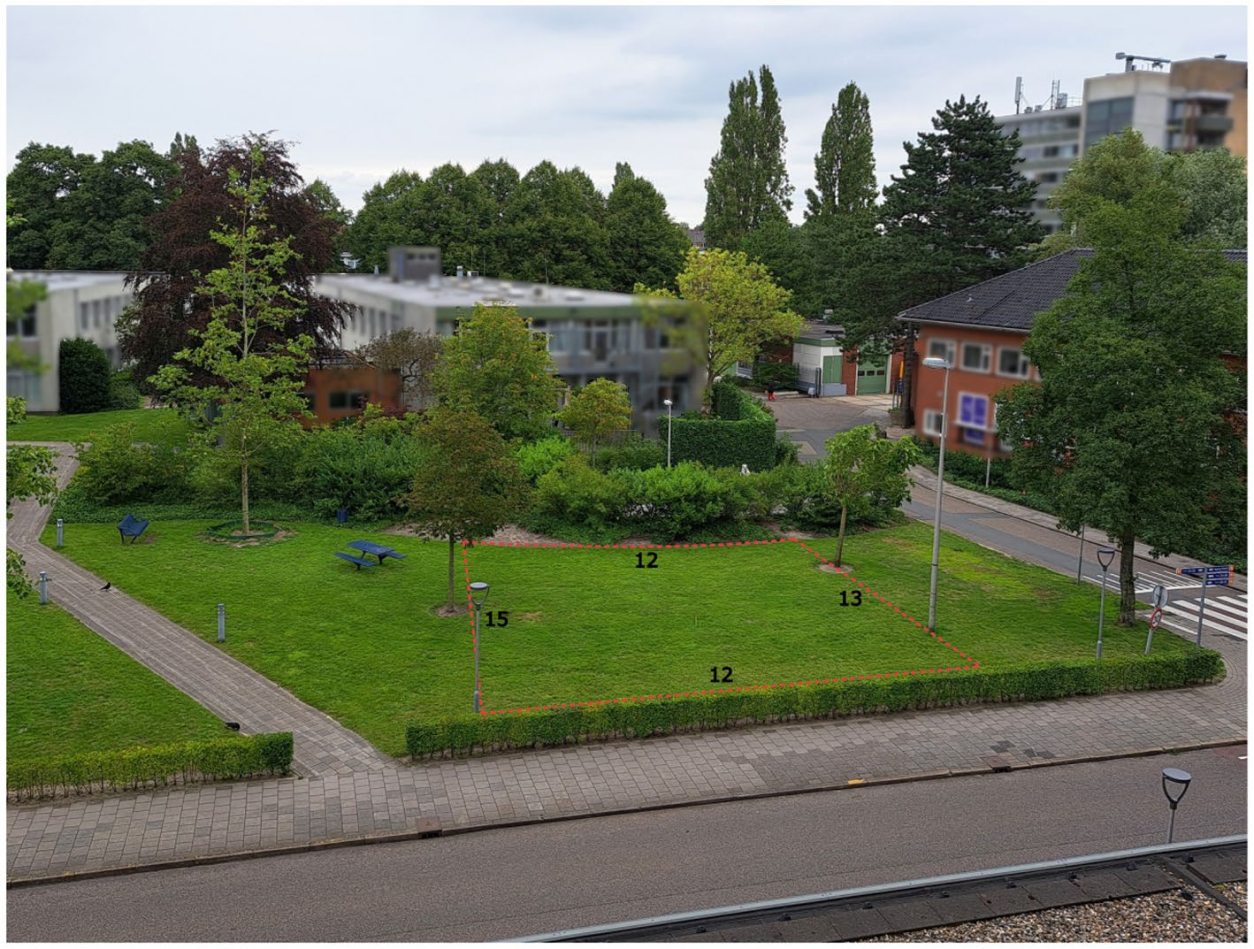
The grass plot on which participants in the in-situ group designed their configurations. The dotted lines indicate the area in which the participants were allowed to place their designs. The lengths of these borders are presented in meters.

All participants first visited the plot outside (see Figure 1). This was necessary to control for the possibility that some participants in the map group were and others were not familiar with the public grass plot and its dimensions. Moreover, architects often also first visit the site they will design for. Following the site visit, the in-situ group stayed on the grass plot to create their configuration on the spot. The map group was instructed to create the configuration on a computer inside the adjacent building.

#### 2.2.1 in-situ group

Participants were informed about the researchers’ interest to design stepping-stones landscapes that would be attractive for people to move in. To that end, participants were asked to create a stepping-stones configuration for themselves in which they would like to step or jump from stone to stone without touching the ground. They were instructed to use six stones (diameter = 55 cm, height = 3 cm, weight = 5.2 kg) and to place their design between the trees, lanterns and grass borders as shown in Figure 1. Participants were not allowed to incorporate the trees and lanterns in their design, stack the stones, or step onto the stones while designing.

By placing six stones, one creates 15 different gap widths.^2^ Obviously not all of these gap widths are necessarily crossable for the participant (e.g., the distance between the farthest stones in a created configuration). Hence, after the design was completed, an experimenter asked for each of the 15 distances between the stones if the participant perceived it as a crossable gap width. Only the gaps that were perceived as being crossable were included in the analysis (see also Jongeneel et al., 2015). Furthermore, we measured leg length by subtracting participant’s sitting height from their standing height (see Warren, 1984). Both the gap widths and the participants heights were determined with a tape measurer.

#### 2.2.2 Map group

**Figure 2:**
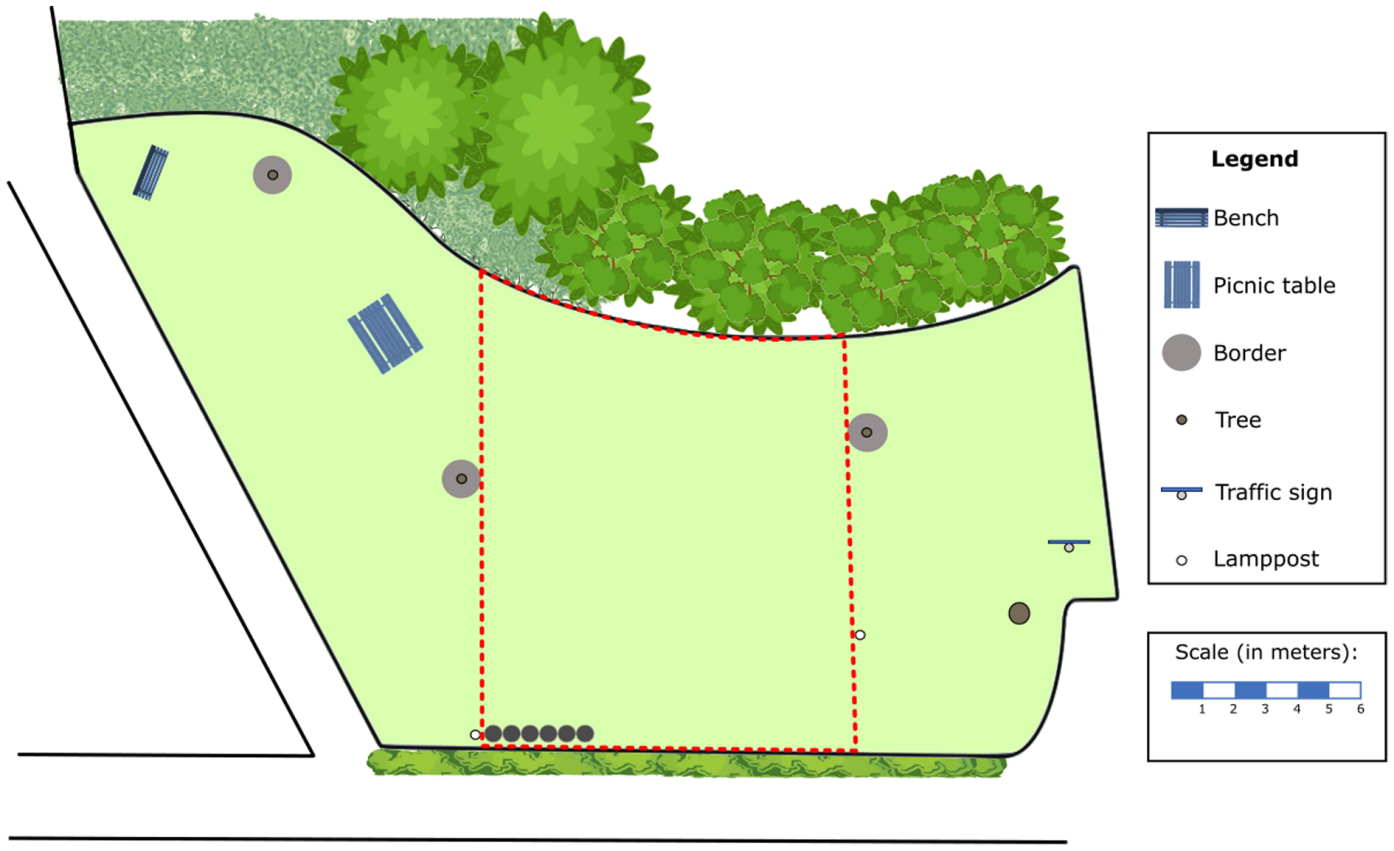
The map of the grass plot on which the map group designed their configurations. The dotted lines indicate the area in which the participants were allowed to place their designs. These borders were not presented to the participant as dotted lines, but pointed out by an experimenter. The legend was presented to participants in Dutch.

The map group received the same background information and almost the same instructions as the in-situ group. However, participants in the map group were instructed to create the configuration on a 2D map of the grass plot, presented in PowerPoint 2016 (see Figure 2). To create the stepping-stones configuration on this map, participants could drag six 2D circles to the desired positions. The circles were scaled to the diameter of the in-situ stones. After the instructions, participants could have one last look on the field through a window (providing a view similar to Figure 1) to make sure that the symbols in the legend of the map were clear. We removed all grid-and guidelines, and made sure that the 2D stones could be dragged in a fluid movement by unticking *snap to grid* in the settings for grids and guidelines. The remainder of the procedure was equivalent to that of the in-situ group.

### 2.3 Data analysis

The gap widths created by the map group were measured using Inkscape (version 1.2.2) and converted to the associated real-life distances. For both groups, we only analyzed the gap widths that a participant perceived as crossable. Using SPSS (version 28), hierarchical cluster analysis (furthest neighbor method) on the Euclidian distances was conducted to determine which of the perceived crossable gap widths were considered to be different or similar. In line with Jongeneel et al. (2015), we used 10% of a configuration’s average gap width as the cut-off point. In other words, within a configuration, we clustered the perceived crossable distances that differed less from each other than 10% of the average gap width—these distances were considered to be similar. The number of derived clusters were taken to represent the number of different gap widths created by a participant.

## 3 Results

**Figure 3:**
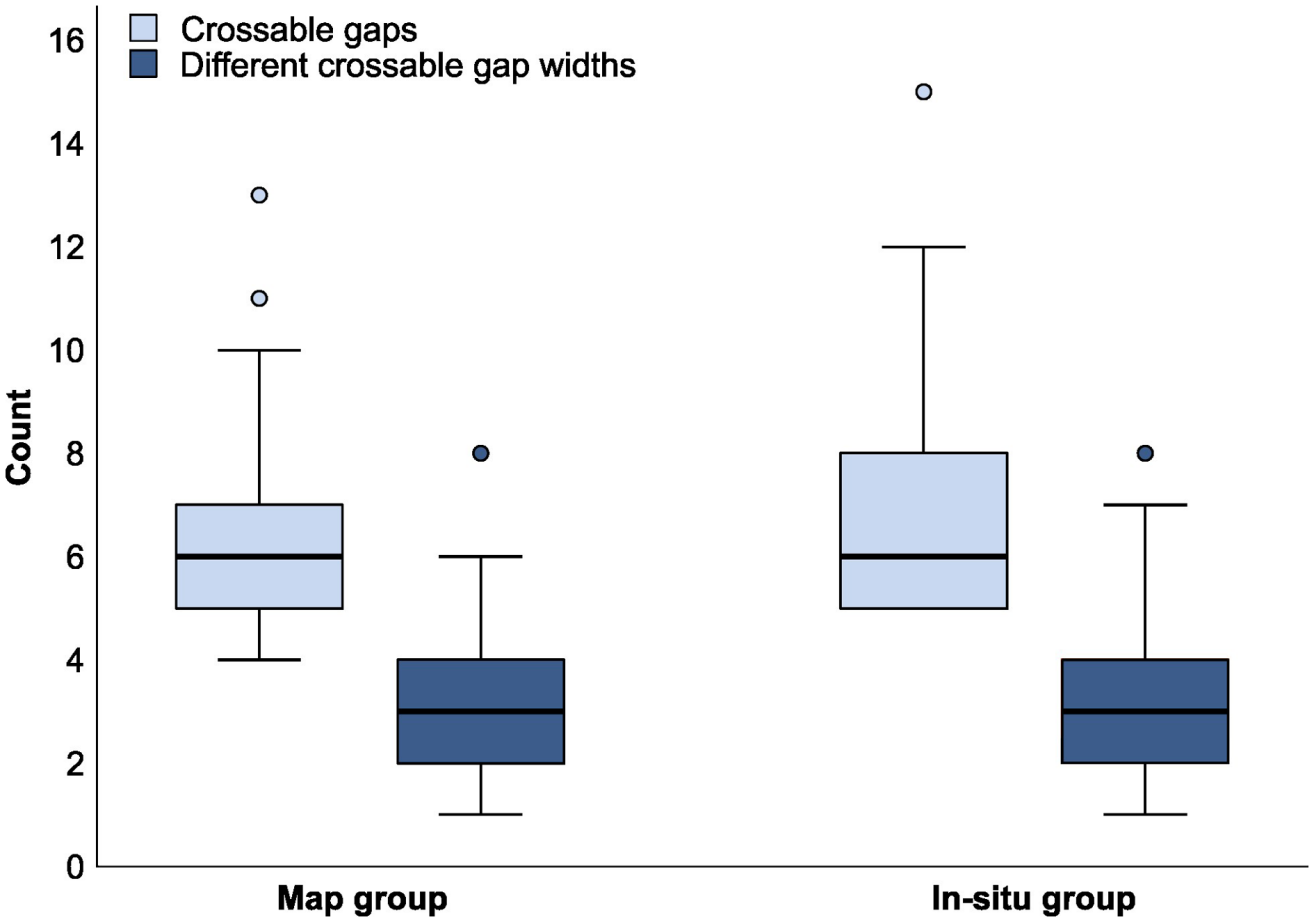
Boxplots of the total number of perceived crossable gaps and the number of different crossable gap widths after hierarchical cluster analysis for both the map and in-situ group. The horizontal, thicker lines within each box present the median values, the boxes present the interquartile range (25th to 75th percentile), the whiskers present the minimum and maximum gap widths within 1.5 interquartile range, and the dots represent outliers of gap widths that were included in the analysis.

Figure 3 shows for both groups the number of perceived crossable gaps and the number of different crossable gap widths (i.e., the number of derived clusters). Both groups created variation in gap widths—75% of the participants in the in-situ group and 63% of the participants in the map group created three or more different distances to cross. Shapiro–Wilk test of normality revealed that there was no normal distribution within groups regarding the number of perceived crossable gaps (*ps* < .001) and the number of different crossable gap widths (*ps* < .001). Although this might not harm the robustness of an independent *t*-test (because of the relatively large sample size; see Sawilowsky and Blair, 1992), we nevertheless opted for using a Mann–Whitney-U test.^3^ No significant differences were found between the groups in the number of indicated crossable gaps (*U* = 2123, *z* = −0.571, *p* = .568, *r* = .049) and in the number of different gap widths after hierarchical cluster analysis (*U* = 2033, *z* = −0.974, *p* = .330, *r* = .084). This suggests that the created degree of standardization was not significantly affected by the perspective on a design.

## 4 Discussion

While architects often design standardized playgrounds and outdoor fitness areas, recent research studying stepping-stones landscapes found that children, young adults and older adults create nonstandardized configurations (Jongeneel et al., 2015; Jeschke et al., 2020). We hypothesized that this difference could be explained by the fact that landscape architects generally design on a 2D map while the participants designed the configurations in situ (see also Olwig, 1990; Withagen and Caljouw, 2017). To test this, we examined the number of different (crossable) gap widths created by participants who designed a stepping-stones configuration in situ and participants who designed the configuration on a map behind a computer. Contrary to our hypothesis, we found no indications that designing in 2D leads to more standardized configurations than designing in situ. Non-architects not only create nonstandardized configurations in real-life, but also created nonstandardized configurations on a 2D map. In the remainder of the discussion, we will explore two other possible hypotheses that could account for the differences between architects and users, and that await testing in future studies.

One possible explanation for the different attraction to standardization could be that architects might be more esthetically motivated than the studied participants. That is, standardization generally leads to visual structure and balance, features found to be esthetically appealing in images (Wilson and Chatterjee, 2005; Hübner and Fillinger, 2016; Van Geert and Wagemans, 2021; Wu et al., 2023; see also Footnote 1; but see Van Geert and Wagemans, 2020). Indeed, it is not unlikely that architects are more concerned about such esthetics in their designs than non-architects (Olwig, 1990; Withagen and Caljouw, 2017). For example, recently our research group was consulted by landscape architects who aimed to create fun stepping-stones landscapes based on our findings so far. We explained to the architects that variation in gap widths was a prominent factor that participants of all ages preferred, and we proposed some drafts. The landscape architects were enthusiastic and adopted the drafts almost completely in the final designs. However interestingly, they still slightly tweaked the stones into a more orderly pattern than we advised (with less gap-width variation), reasoning that the appearance of the configuration would otherwise be “too messy” for the streetscape. Participants in our research, on the other hand, were possibly less concerned about such esthetic-related factors, resulting in more nonstandardized landscapes.

Another hypothesis is that architects are trained to work with standardized measures of architectural elements. Indeed, Imrie (2003) interviewed several architects and architectural course tutors in the United Kingdom and noted that “most degree schemes use a range of textbooks, such as the Metric Handbook (Adler, 1999) and Neufert and Neufert’s (2000) Architects’ Data, that do little to challenge the dominance of geometrical discourses” (Imrie, 2003, p. 54). The books, like many other design manuals (e.g., Panero and Zelnik, 1979; Bielefeld, 2018), present extensive lists of standard architectural dimensions that are based on the *average* human proportions and ranges of motion. Indeed, it is truly challenging to take into account the wide variety of action capabilities of a target group. Consequently, it is a “fail-safe option” (Imrie, 2003, p. 56) to base a measure within a design on a recommended, standard dimension. That is, whereas the examined non-architects could seek for a variety of action possibilities within their range of action capabilities, architects might be early-on primed to design with certain predefined measures of target groups recommended by design manuals.

Future research is needed to further scrutinize these hypotheses. Regardless, when aiming to design physical-activity-inducing environments, one should be aware of the criticism against standardized measures (e.g., Olwig, 1990; Nebelong, 2004; Withagen and Caljouw, 2017). First, humans vary in their bodily dimensions and action possibilities. Hence, to afford challenging and/or fun playgrounds and outdoor fitness areas to a variety of people, one should create a variety of gap widths (e.g., Withagen and Caljouw, 2017). Secondly, nonstandardized playground designs are better equipped to facilitate versatile movement than standardized playground design. And according to the variability-of-practice hypothesis, motor skills develop better when practiced in variable ways (e.g., Schmidt, 1975; Schmidt and Lee, 2003; Schöllhorn et al., 2010; Chow et al., 2011). Finally, and perhaps most important to attract people to the sites, the created designs in the present study once again underpin that the target users seem to prefer nonstandardized dimensions over standardized ones (see also Jongeneel et al., 2015; Sporrel et al., 2017; Jeschke et al., 2020, 2022, 2023).

## Data availability statement

The raw data supporting the conclusions of this article will be made available by the authors, without undue reservation.

## Author contributions

AJ: Writing – original draft, Writing – review & editing. SC: Writing – original draft, Writing – review & editing. FZ: Writing – original draft, Writing – review & editing. RW: Writing – original draft, Writing – review & editing.

## Ethics statement

The studies involving humans were approved by the local ethics committee (CTc, UMCG). The studies were conducted in accordance with the local legislation and institutional requirements. The participants provided their written informed consent to participate in this study.

## Footnotes

^1^Note that participants in the cited studies judged the configurations’ esthetics while standing next to them. Interestingly, a recent pilot study showed that when participants looked at images depicting stepping-stones configurations from a top-view perspective (the perspective from which many architects design), they find the symmetrical configurations significantly more beautiful than non-symmetrical ones. However, when judging images depicting the same configurations from a more everyday perspective (as when standing next to them), these differences appeared to vanish or even to flip. In fact, as the cited studies revealed, participants may then find nonstandardized configurations slightly more esthetic.

^2^Placing *n* stones creates ((*n*−1)/2)**n* gaps (see Jongeneel et al., 2015).

^3^An independent samples *t*-test also revealed no significant differences between groups in the total number of created gap widths (*t*(132) = −0.775, *p* = 0.440, *d*=0.134) and in the number of different gap widths (*t*(132) = −0.785, *p* = .434, *d*=0.136).
